# Genomic and Metabolomic Profile Associated to Microalbuminuria

**DOI:** 10.1371/journal.pone.0098227

**Published:** 2014-06-11

**Authors:** Vannina G. Marrachelli, Daniel Monleon, Pilar Rentero, María L. Mansego, Jose Manuel Morales, Inma Galan, Remedios Segura, Fernando Martinez, Juan Carlos Martin-Escudero, Laisa Briongos, Pablo Marin, Gloria Lliso, Felipe Javier Chaves, Josep Redon

**Affiliations:** 1 Metabolomic and Molecular Image Lab, Health Research Institute, INCLIVA, Valencia, Spain; 2 Genotyping and Genetic Diagnosis Unit, Health Research Institute, INCLIVA, Valencia, Spain; 3 Department of Nutrition, Food Science and Physiology. University of Navarra, Pamplona, Spain; 4 CIBERObn, Health Institute Carlos III, Madrid, Spain; 5 Hypertension Unit, Internal Medicine, Hospital Clinico, Valencia, University of Valencia, Spain; 6 Internal Medicine, Hospital Rio Hortega, Valladolid, Spain; 7 CIBERDem, Health Institute Carlos III, Madrid, Spain; Westfaelische Wilhelms Universitaet, Germany

## Abstract

To identify factors related with the risk to develop microalbuminuria using combined genomic and metabolomic values from a general population study. One thousand five hundred and two subjects, Caucasian, more than 18 years, representative of the general population, were included. Blood pressure measurement and albumin/creatinine ratio were measured in a urine sample. Using SNPlex, 1251 SNPs potentially associated to urinary albumin excretion (UAE) were analyzed. Serum metabolomic profile was assessed by ^1^H NMR spectra using a Brucker Advance DRX 600 spectrometer. From the total population, 1217 (mean age 54±19, 50.6% men, ACR>30 mg/g in 81 subjects) with high genotyping call rate were analysed. A characteristic metabolomic profile, which included products from mitochondrial and extra mitochondrial metabolism as well as branched amino acids and their derivative signals, were observed in microalbuminuric as compare to normoalbuminuric subjects. The comparison of the metabolomic profile between subjects with different UAE status for each of the genotypes associated to microalbuminuria revealed two SNPs, the rs10492025_TT of *RPH3A* gene and the rs4359_CC of *ACE* gene, with minimal or no statistically significant differences. Subjects with and without microalbuminuria, who shared the same genotype and metabolomic profile, differed in age. Microalbuminurics with the CC genotype of the rs4359 polymorphism and with the TT genotype of the rs10492025 polymorphism were seven years older and seventeen years younger, respectively as compared to the whole microalbuminuric subjects. With the same metabolomic environment, characteristic of subjects with microalbuminuria, the TT genotype of the rs10492025 polymorphism seems to increase and the CC genotype of the rs4359 polymorphism seems to reduce risk to develop microalbuminuria.

## Introduction

Increased urinary albumin excretion (UAE) is today considered an expression of abnormal endothelial permeability and an integrated marker of cardiovascular and renal risk [1], [2], [3]. Largely studied in diabetes, its importance as a risk factor in hypertension and in the general population has been established in the last years [4]. A large number of cross-sectional and follow-up studies have established the relationship between UAE and blood pressure (BP) as well as insulin levels [5]. The importance of other casual factors such as obesity and smoking contribute, to a minor degree, to the increment of UAE. The role of genetic factors in the risk to develop an increment in UAE has been a matter of controversy. The relationship between the risk of UAE and polymorphisms of candidate genes was initially described [6]. A recent review about the potential genetic factors described the most relevant information available but no firm conclusions can be established [7]. Furthermore, a GWAS analysis of a very large number of subjects did not find genetic traits associated with UAE, although how the phenotype was assessed had all the potential for inaccuracy [8].

Intermediate mechanisms have also provided some insights into the increment of UAE and its association with high cardiovascular and renal risk. Inflammation [9] and oxidative stress [10] have been associated to microalbuminuria as intermediate mechanisms. Other potential associations can be explored by using the metabolomic profile. The study of small-molecule metabolites in biological fluids with NMR spectroscopy (^1^H-NMR spectra), a fast and reproducible technique, may be useful for understanding metabolic imbalances and for detecting previously unsuspected links to pathological conditions [11], [12]. Recently studies using metabolomics have been applied to identify a discriminatory metabolite profile in a large number of diseases [13], [14], [15]. Up to now no information about the link between metabolomic profiles and UAE in the general population has been provided. A further step in identifying the factors related to the risk to develop microalbuminuria could be to take advantage of combining data provided from genomic and metabolomic analyses, the goal of the present study in a general population.

## Methods

### Study Population

The study was carried out in subjects from a population-based study in which the selection criteria and methodology have been previously described [16]. Briefly, the sample included individuals older than 18 years in the absence of serious concomitant disease or psychiatric disorder, which could interfere with the study. All the subjects included in the study were Caucasian, living in an area with a low immigration rate. To be representative of the general population, investigators calculated the sample size by using local public resources and finally 1502 subjects were included. From all the patients studied, 1217 with a high genotyping call rate were analysed. The Ethical Committee of the Hospital Rio Hortega, Spain approved the study and all the participants gave written agreement to participate. Participants gave their informed consent to use their blood samples for genetic studies.

### Assessment of Cardiometabolic Risk Factors

The study included the assessment of anthropometric measurements, blood pressure, glycaemia, lipid profile and smoking status as well as personal and familial information about cardiovascular risk factors and diseases. Weight was assessed with precise scales while the individuals were without shoes and with light clothing. Height was determined in a similar way. Body mass index (BMI) was calculated using the following formula “weight (Kg)/height^2^ (m)”. Glucose and lipid profile was measured in blood samples obtained with a mean of 3 hours fasting (range 0–17). Basic serum biochemistry and lipid profile (total cholesterol, HDL cholesterol and triglycerides) were measured in Hitachi 917 autoanalyzer (Boehringer, Germany). Type 2 diabetes was defined when fasting glucose was equal to or higher than 126 mg/dl prior diagnosis or if treatment for diabetes. When a patient had glucose >110 mg/dl in the fasting state or >126 mg/dl in non-fasting state an oral glucose tolerance test was performed. This test was interpreted according to the recommendations of the expert panel for the diagnosis and classification of DM2 [17]. Blood pressure was measured using a mercury sphygmomanometer following the recommendations of the British Hypertension Society. Systolic BP (SBP) and diastolic BP (DBP) were the average of 3 readings measured at 5-minute intervals. The GFR was estimated (EGFR) by the MDRD abbreviated formula [18].

### Urinary Albumin Excretion Measurement

Urinary albumin excretion was measured in morning urine collections using a nephelometric immunoassay (Behring Institute). For each patient, the albuminuria was considered as the mean value obtained from the morning spot urine samples and expressed as albumin (µg)/creatinine (mg) ratio (ACR). Microalbuminuria (mALB) was defined as ACR≥30 µg/mg. The coefficient of reproducibility for the UAE measurement in our laboratory was intra-assay 2%; interassay 6% and intraindividual 12%.

### Single-Nucleotide-Polymorphism Selection and Genotyping

One thousand two hundred and fifty one single nucleotide polymorphisms (SNP) potentially associated to UAE were selected based on a bibliography search and the SYSNPS program (www.sysnps.org) [19]. SYSNPs use the following databases and software versions: Ensembl 61, HapMap release 28 and Haploview 4.2. Minor allele frequency (MAF) of all the SNPs in the selected gene was analysed in CEPH (Utah residents with Northern and Western European Ancestry) population. Organizing selected polymorphisms into LD bins with pairwise r^2^>0.8, SNPs with MAF>0.1, located in all their functional positions (upstream and downstream, 5′ and 3′-untranslated region, exons); and previous positive findings in association studies were selected. These SNPs include genes involved in lipid metabolism, oxidative stress, mitochondrial respiratory chain, renin-angiotensin system and other biological processes as being systems previously associated to UAE. Genotyping was carried out using an oligo-ligation assay (SNPlex, Applied Biosystems, Foster City, California, USA) following the manufacturer’s instructions.

### NMR Spectroscopy

Eighty-two microliters of D_2_O were added to 418 µl of blood serum and placed in a 5-mm NMR tube. ^1^H NMR spectra were recorded using a Bruker Avance DRX 600 spectrometer (Bruker GmbH, Rheinstetten, Germany). Samples were measured at 37°C. Nominal temperature of the sample was kept at 37°C. A single-pulse pre-saturation experiment was acquired in all samples. The spectra were referenced using the doublet of Alanine at 1.478 ppm. The chemical shift region including resonances between 0.50 and 4.70 parts per million of spectrometer frequency (ppm) was investigated. The spectra were normalized to total aliphatic spectral area to eliminate differences in metabolite total concentration. The spectra were binned into 0.01 ppm buckets and mean centered for multivariate analysis. Consequently, to minimize noise, the spectra were reduced to 51 regions based on its metabolites enrichment. Peak area integration was used to calculate the relative contributions. Signals belonging to selected regions were quantified using semi-automated in-house MATLAB 6.5 (The MathWorks Inc., Natick, Massachusetts) integration and peak-fitting routines. Quantification was assessed for spectral regions containing contributions of a single or at most two metabolites. For the remaining metabolites, quantification was compromised due to low signals and/or overlapping.

Reproducibility of NMR spectroscopy was tested by superposition of normalized spectra of blood serum. Annotation of significant metabolites was achieved through the identification of full spin systems from analysis of two-dimensional NMR experiments including homonuclear correlation spectroscopy (TOCSY) and heteronuclear single quantum correlation spectroscopy (HSQC), which provides statistical correlations between NMR variables suggesting structural or biological connectivity. Metabolite assignment procedure exploited knowledge from academic spectral databases such as HMDB [20] as well as proprietary databases (Chenomx NMR Suite 4.5).

Chemometric statistical analyses were performed using in-house MATLAB scripts and the PLS Toolbox (Eigenvector Research, Inc.). A principal component analysis (PCA) for each serum was firstly performed corresponding to an unsupervised multivariate data reduction routine, which serves to evaluate the data distribution and intersample similarities quickly (e.g., clusterings and outliers). After the PCA analysis, a partial least-squares discriminant analysis (PLS-DA) is usually used to build a statistical model that optimizes the separation between the two groups (subjects with and without microalbuminuria). The multivariated chemometric models were cross-validated with 10-fold Venetian blind cross-validation; in each run 10% of the data were left out of the training and used to test the model. The whole cross validation process was run 10 times. The results of cross validation were evaluated by the Q2 (R2CV) and RMSCV parameters. Q2 is the averaged correlation coefficient between the dependent variable and the PLS-DA predictions and provides a measure of prediction accuracy during the cross-validation process (higher values mean better prediction). Root Mean Square Error of Cross-Validation (RMSCV) was calculated as an adequate measurement of over fitting.

### Statistical Analysis

All values are expressed as mean ± SD. The χ^2^ goodness-of-fit test was used to compare the distribution of the study population. Genotypes and allele frequencies were calculated for every SNP. The Hardy-Weinberg equilibrium was sought by a χ^2^-distribution with one degree of freedom. Those SNPs that were not in Hardy-Weinberg equilibrium and did not have more than 90% of genotyping were excluded from the subsequent analysis. The Hardy-Weinberg equilibrium was calculated using PLINK (http://pngu.mgh.harvard.edu/~purcell/plink/). The association of microalbuminuria with each polymorphism was performed using PLINK by logistic regression models. Urinary albumin excretion was log transformed (logUAE) and associations were tested by linear regression models, adjusted by age, sex, BMI, Systolic BP and fasting glucose. A Holm-Bonferroni method was used to correct the problem of multiple testing. Holm-Bonferroni represents a simple test and a stepwise algorithm more powerful than the Bonferroni correction. Our finally selection of SNPs was made based on the Holm-Bonferroni results and the differences in metabolomics profile. This double criterion further restricts our results to meaningful genotypes associated to differential expression of UAE.

The metabolomic profiles of patients with and without microalbuminuria were compared. Among all the metabolites measured, those with the highest contribution to the PLS-DA discrimination model were selected for further analysis. We explored the association between a metabolic profile and genetic variants using these selected metabolites. We aimed to detect genotypes showing the lowest metabolic differences with microalbuminuria. For individuals with the corresponding SNPs, we calculated the average metabolic level and standard deviation for each individual metabolite in microalbuminuria and no microalbuminuria normalized with respect groups stratified by SNPs. For each polymorphism normalized to the same differences at global levels, irrespective of genotype. Differences in the 26 metabolite values for each SNP in subjects with and without microalbuminuria of each genotype were calculated.

Finally, the metabolic profile and the most relevant metabolites of each genotype and allele were compared between normoalbuminuric and microalbuminuric subjects. The data was adjusted for the potential confounders in the study population age, sex, BMI, type 2 diabetes, and SBP. Statistical analyses were performed using the IBM SPSS Statistics 19 software.

## Results

### General Characteristics of the Study Population

A total of 1231 subjects, mean age 54±19 of both sexes, 50.6% men, were included in the study. Albumin creatinine ratio was <30 mg/g in 1092 (89%), and >30 mg/g in 81 (11%) subjects. The main characteristics of the study population grouped by UAE status are present in Table 1.

**Table 1:** General characteristics of the study population grouped by urinary albumin excretion.

	No Malb	Malb	P
Number of samples	1150	81	
Sex (M/F)	582 (93.4)/568 (93.4)	41 (6.6)/40 (6.6)	1
Age (years)	53±19	67±18	0.052
BMI (kg/m^2^)	26±6	28±5	0.026
SBP (mm Hg)	129±21	153±29	7.3 E-10
DBP (mm Hg)	79±12	83±13	0.002
Glycemia (mg/dl)	92±20	104±29	9.5 E-7
Creatinine (g/24 h)	112±56	118±64	0.315
Total Cholesterol (mg/dl)	201±38	202±42	0.846
LDL (mg/dl)	114±35	114±34	0.895
HDL (mg/dl)	52±14	47±14	0.040
LogTG (mg/dl)	2.18±0.23	2.26±0.20	0.002
Diabetes mellitus 2	77 (6.7)	22 (27.2)	<0.0001
Hypertension	450 (39.1)	64 (79.0)	<0.0001
Metabolic syndrome	237 (20.6)	47 (58)	<0.0001
Obesity	258 (22.4)	31 (38.3)	<0.0001
HTN treatment	203 (17.7)	36 (44.4)	<0.0001
DM treatment	42 (3.7)	12 (14.8)	<0.0001
HCT-TG treatment	72 (6.3)	11 (13.6)	0.019

*() percentage*.

### UAE and SNPs Polymorphism

From the total 1251 SNPs tested, fourteen polymorphisms on 12 genes were significantly associated with LogUAE: *FADS2* (rs174611, rs174577 and rs174616), *GNB3/LEPREL2* (rs 3759347), *CAT* (rs511895), *FADS1* (rs174546), *RPH3A* (rs10492025), *BBS2/MT4* (rs1566441), *MT3* (rs11644094), *GNB3* (rs1129649), *APOA5* (rs662799), *ACE* (rs4359), *FTO* (rs8044769) and *APOC3/APOA1/APOA4* (rs651821). The main characteristics of the SNPs and the degree of association are shown in Table 2. There is a predominance of associated SNPs in the chromosome 11, 12 and 16, mainly in genes related with metabolic factors, oxidative stress, G-protein, obesity and angiotensin converting enzyme inhibitor.

**Table 2:** SNPs associated to UAE in the general population.

Chr	SNP*	model	Gene	Gene description	Location	Beta	SE	*p* value**	Power (%)
11	rs174611	ADD	*FADS2*	fatty acid desaturase 2	INTRON	0,1759	0,05069	0,0005402	94,62
11	rs174577	ADD	*FADS2*	fatty acid desaturase 2	INTRON	0,1755	0,04799	0,0002677	96,23
11	rs174616	ADD	*FADS2*	fatty acid desaturase 2	INTRON	0,0904	0,04436	0,04177	54,32
11	rs174546	ADD	*FADS1*	fatty acid desaturase 1	INTRON	0,1562	0,04914	0,001526	89,84
11	rs511895	ADD	*CAT*	Catalase	INTRON	−0,1369	0,04774	0,00421	84,77
11	rs662799	ADD	*APOA5*	apolipoprotein A-V	INTRON	0,2792	0,0952	0,003431	82,39
11	rs651821	DOM	*APOC3/APOA1/APOA4*	apolipoprotein	INTERGENIC	0,2649	0,09611	0,005952	77,78
12	rs3759347	ADD	*GNB3/LEPREL2*	G protein beta polypeptide3/leprecan-like 2	INTRON/INTRON	0,1643	0,0493	0,0008897	92,89
12	rs10492025	ADD	*RPH3A*	rabphilin 3A homolog (mouse)	INTRON	0,1411	0,04879	0,003906	84,73
12	rs1129649	ADD	*GNB3*	G protein beta polypeptide 3	p.Ile685Thr	0,1246	0,04766	0,009086	76,98
16	rs1566441	ADD	*BBS2/MT4*	metallothionein 4	INTERGENIC	−0,1302	0,04611	0,004825	84,11
16	rs11644094	REC	*MT3*	metallothionein 3	INTRON	0,1121	0,04535	0,01365	72,27
16	rs8044769	DOM	*FTO*	fat mass and obesity associated	INTRON	−0,1306	0,04505	0,003821	84,66
17	rs4359	ADD	*ACE*	angiotensin I converting enzyme I(peptidyl-dipeptidase A) 1	INTRON	−0,1316	0,04679	0,004994	83,51

*dbSNP; adjusted by age, sex, BMI, Systolic BP and fasting glucose.

### UAE and Metabolomic Profile

**Figure 1:**
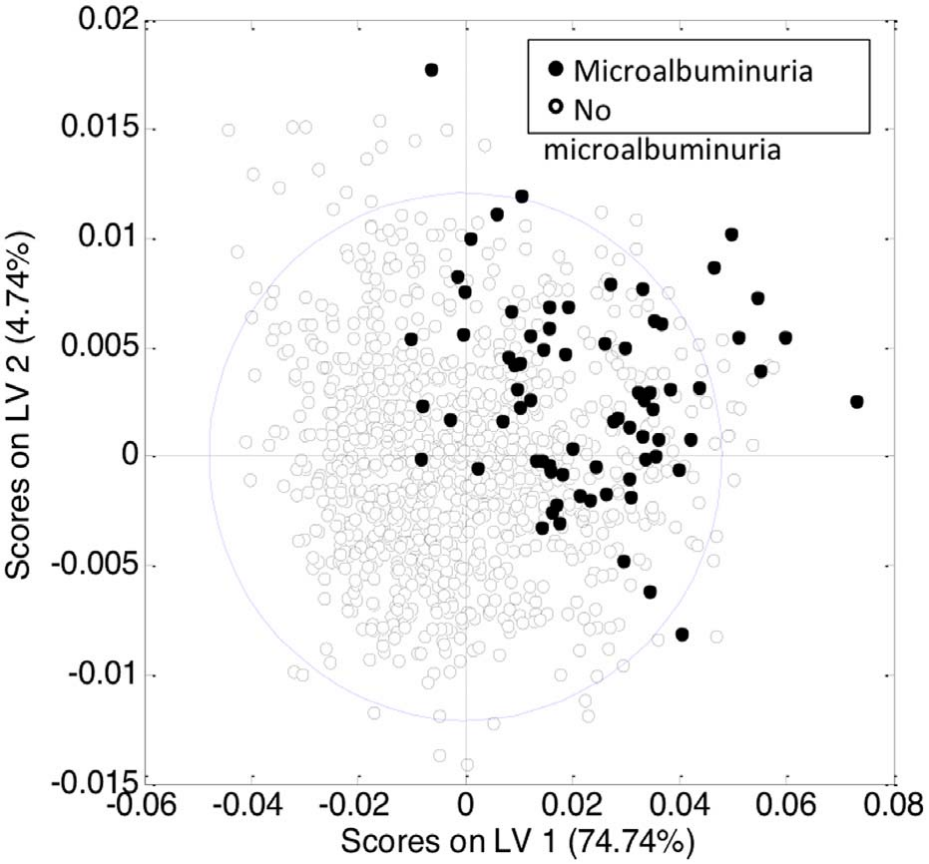
PLS-DA model scores plot for discrimination between patients without (open circles) and with microalbuminuria (close circles) based on the NMR spectra of blood serum of the entire cohort.

**Figure 2:**
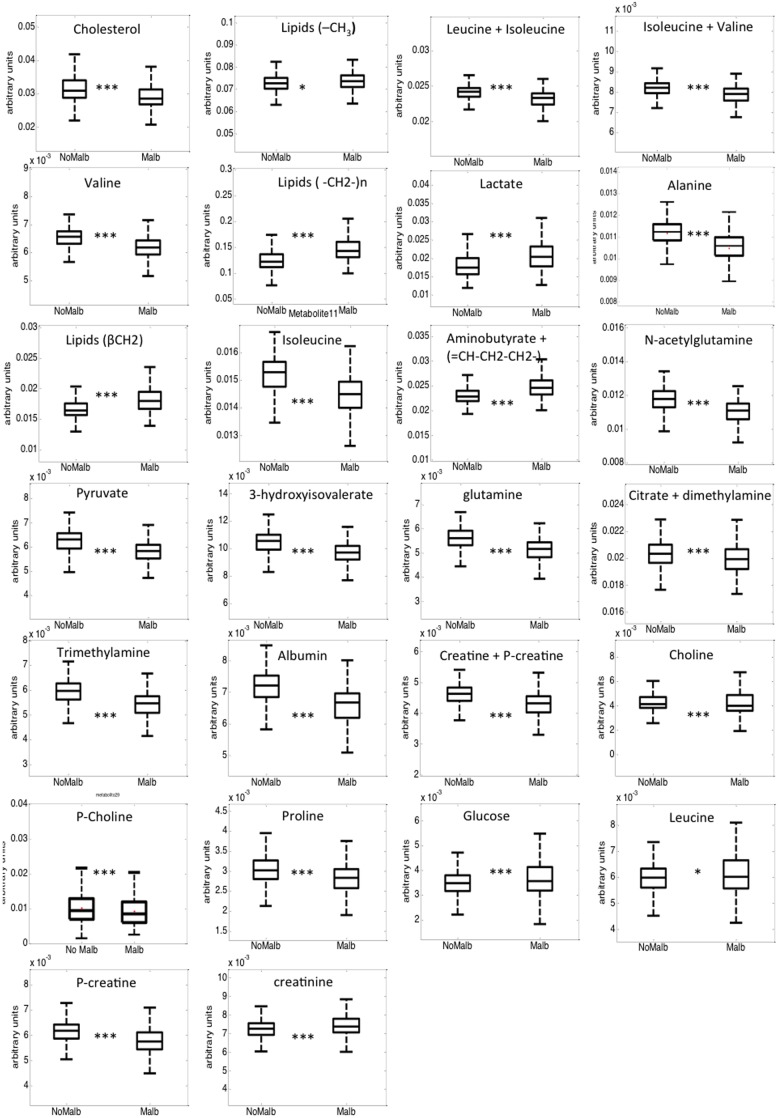
Box plots showing metabolite relative levels in blood serum of subjects without microalbuminuria (box in the left) and with microalbuminuria (box in the right). Boxes denote interquartile range, lines denote median, and whiskers denote tenth and ninetieth percentiles. Levels are expressed as area of the metabolite of interest divided with respect total aliphatic spectral area. *, p-value<0.01; ***, p-value<0.00001.

Principal component analysis (PCA) was initially performed with the normalized peak areas obtained from all the samples to evaluate the quality of sample analysis and to view the holistic distribution, clustering, and outlier of samples. The PCA scores plot shows that most of the samples in the study are tightly clustered in a small area, indicating that the current protocol is reliable and thereby the variance derived from metabolomic analysis can be ignored at the following data analysis. Then, partial least squares discriminant analysis (PLS-DA) was applied (Figure S1). The PLS-DA model showed goodness of fit (RMSCV = 0.36), adequate model predictability (Q2 = 0.25), and fairly good capability to explain the metabolic variation between normoalbuminurics and those with microalbuminuria. After spectral integration, differences were observed among subjects with and without microalbuminuria (Figure 1). As shown in Table 3, the differential endogenous compounds detected included mitochondrial metabolism (citrate), extra mitochondrial metabolism (glucose, piruvate, lactate, creatinine, creatine, creatine phosphate) and several amino acids and their derivative signals (such as proline, glutamine, N-acetylglutamine, alanine). Among these, branched amino acids (valine, isoleucine and 3-hydroxyisovalerate) exhibited a relatively high statistical significance. We also detected numerous fatty acid signals, (FA-CH3, FA-CH2-CH2CO, FA-CH2-CH3), as well as signals from cholesterol, choline and phosphocholine, aminobutyrate, dimetylamine, trimethylamine, and albumin (Figure 2).

**Table 3:** Metabolite relative levels in serum patients with and without microalbuminuria.

Metabolites	ppm	Nomicroalbuminuria	Microalbuminuria	P-value
**Cholesterol**	0.60–0.75	3.17±0.44	2.95±0.44	<0,001
**Lipid (–CH_3_)**	0.80–0.90	7.26±0.39	7.35±0.47	0,002
**Isoleucine + Leucine**	0.92–0.97	2.41±0.10	2.31±0.15	<0,001
**Isoleucine + valine**	0.98–1.00	0.82±0.04	0.78±0.06	<0,001
**Valine**	1.03–1.05	0.65±0.03	0.61±0.05	<0,001
**Lipids (-CH_2_-)n**	1.20–1.32	12.59±2.08	14.78±2.91	<0,001
**Lactate**	1.32–1.35	1.83±0.38	2.09±0.45	<0,001
**Alanine**	1.46–1.49	1.12±0.06	1.05±0.08	<0,001
**Lipids (βCH_2_)**	1.55–1.60	1.67±0.16	1.82±0.23	<0,001
**Isoleucine**	1.92–1.97	1.52±0.07	1.44±0.09	<0,001
**4-aminobutyrate + ( = CH-CH_2_-CH_2_-)**	1.98–2.03	2.31±0.18	2.48±0.25	<0,001
**N-acetylglutamine**	2.10–2.15	1.17±0.07	1.10±0.09	<0,001
**Pyruvate**	2.34–2.38	0.62±0.05	0.58±0.06	<0,001
**3-OH-isovalerate**	2.34–2.41	1.05±0.08	0.96±0.09	<0,001
**Glutamine**	2.43–2.47	0.56±0.04	0.51±0.06	<0,001
**Dimetylamine**	2.65–2.80	2.03±0.11	1.99±0.13	<0,001
**Trimethylamine**	2.90–2.95	0.59±0.05	0.54±0.06	<0,001
**Albumin**	2.95–3.00	0.72±0.06	0.66±0.07	<0,001
**Creatine + creatineP**	3.02–3.05	0.46±0.03	0.43±0.05	<0,001
**Choline**	3.18–3.20	0.46±0.03	0.43±0.05	<0,001
**O-phosphocholine**	3.21–3.22	1.02±0.42	0.92±0.37	<0,001
**Proline**	3.32–3.37	0.30±0.04	0.28±0.04	<0,001
**Glucose**	3.45–3.48	0.35±0.07	0.38±0.11	<0,001
**Leucine**	3.68–3.72	0.60±0.07	0.61±0.10	0,015
**Creatine-P**	3.93–3.97	0.61±0.05	0.58±0.08	<0,001
**Creatinine**	4.03–4.08	0.72±0.05	0.75±0.13	<0,001

### Metabolomic Profile, Selected Genotypes and UAE

**Figure 3:**
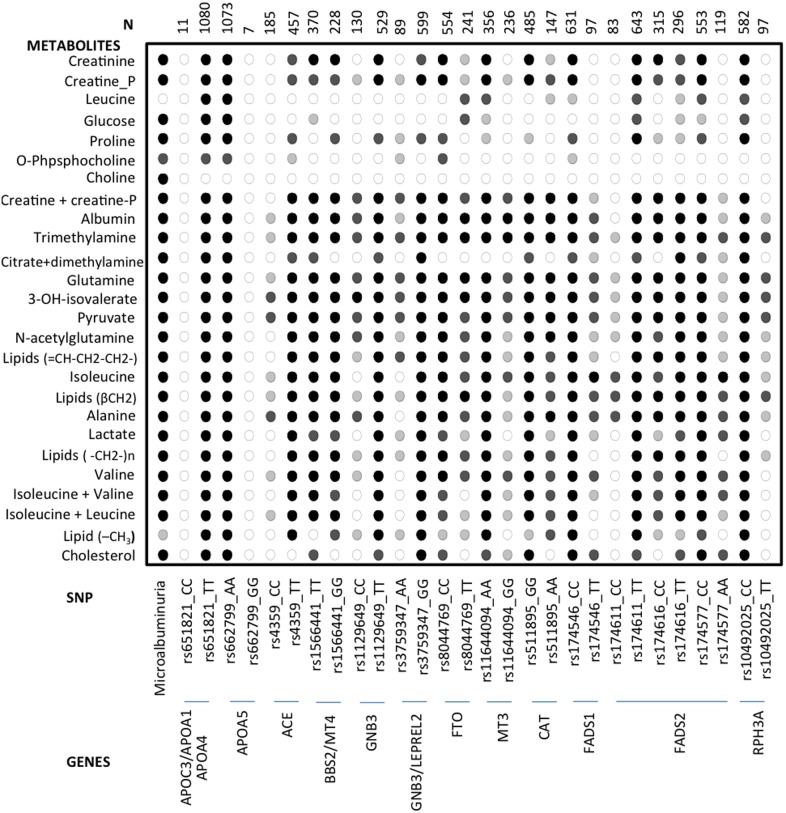
Patterns of statistical significance, calculated as p-values, for the comparison of metabolic profiles between normoalbuminuria and microalbuminuria in the whole population (first column) and in individuals with different SNPs (rest of the columns). (○) p>0.01; (light grey circle) p<0.01; (dark grey circle) p<0.001; (•) p<0.00001. Percent of microalbuminuria in each genotype is included between brackets ().

**Figure 4:**
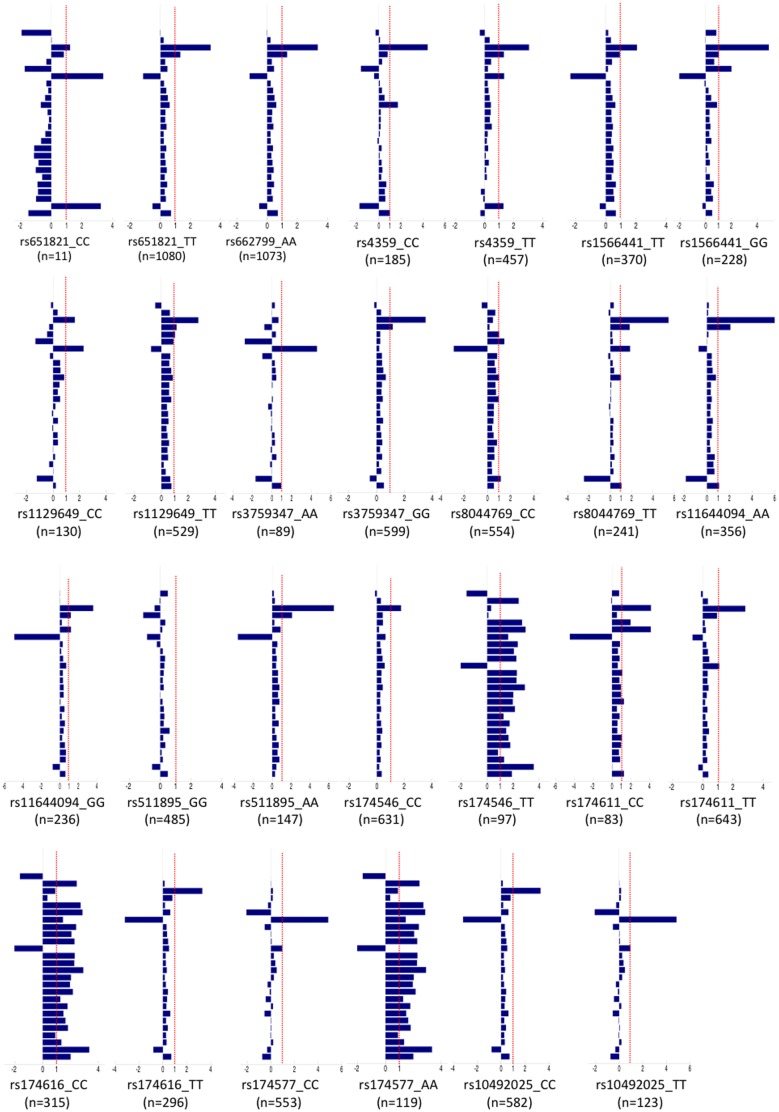
Bar chart showing metabolic differences between microalbuminuria and no microalbuminuria normalized with respect to changes in the entire cohort. The bars represent the difference in the average metabolic levels between microalbuminuria and no microalbuminuria for each SNP divided by the same difference calculated for the entire cohort. SNPs with bars closer to 1 (dotted line) show UAE associated metabolic changes similar to those of the global population (irrespective of genotype). On the other hand, SNPs with bars closer to 0 exhibit minimal or no metabolic changes associated to UAE. Bars with negative values indicate a UAE associated metabolic change opposite to that detected in global population. Metabolites from top to bottom are: creatinine; creatine phosphate; leucine; glucose; proline; phosphocholine; choline; creatine+creatine phosphate; albumin; trimethylamine; citrate+dimethylamine; glutamine; 3-hydroxyisovalerate; pyruvate; N-acetylglutamine; lipids ( = CH-CH2-CH2-)+aminobutyrate; isoleucine; lipids (βCH2); alanine; lactate; lipids (-CH2-)n; valine; valine+isoleucine; leucine+isoleucine; lipids (-CH3) and cholesterol.

The metabolomic profiles of the genotypes of the 14 SNPs associated to log UAE were obtained. In each of these genotypes we compared the values for each individual metabolite between normoalbuminuric and microalbuminuric subjects as a whole and in each individual genotype, Figures 3 and 4. The comparison of the statistical significance patterns revealed two genotypes in two SNPs in which predominate the microalbuminuric profile but with minimal or no statistically significant differences between UAE status, the rs10492025_TT of the *RPH3A* gene and the rs4359_CC of the *ACE* gene. We built PLS-DA models for these individual SNPs to evaluate global metabolic differences between groups (Q2 = 0.09 for rs4359_CC and Q2 = 0.11 for rs10492025_TT). The cross validation parameters and the scores plot of these models shown that discrimination with respect to UAE was worse in these SNPs than in the global analysis.

Then, we looked for the characteristics of the subjects with and without increment in UAE who share the same genotype and the same metabolomic profile (Table S1). Although microalbuminuric subjects have a higher prevalence of hypertension and/or diabetes, those with CC genotype of the rs4359 polymorphism (17 subjects) were older as compared with the total microalbuminuric population (74±6 vs 67±18; respectively p = 0.006). In contrast, among subjects with TT genotype of the rs10492025 polymorphism, those with microalbuminuria (7 subjects), although not statistically different, tend to be younger as compared to total microalbuminuric population (50±18 vs 67±18; p = 0.02). It seems that the CC genotype of the rs4359 polymorphism can act as protector for the development of microalbuminuria while the TT genotype of the rs10492025 polymorphism could facilitates the development of microalbuminuria.

## Discussion

In the present study, we identified a metabolomic profile associated to the presence of microalbuminuria, characterized by an increment in some mitochondrial and extra-mitochondrial metabolism derivate metabolites and fatty acid signals, as well as a decrease in branched amino acids. This microalbuminuric metabolomic profile was also present in normoalbuminuric subjects who share the genotype of two SNPs on the *ACE-I* and the *RPH3A* (rabphilin 3A homolog) genes. We hypothesize that with the same metabolomic environment, individuals sharing the TT genotype of the rs10492025 polymorphism seems to have a higher risk, and those with the CC genotype of the rs4359 polymorphism partially protected from the development of microalbuminuria in the presence of hypertension and or diabetes.

The study was performed in subjects representative of the general population from an area with a low rate of external admission. In this population, the prevalence of microalbuminuria was in agreement with other population-based studies [21]. Microalbuminuria, was associated to the presence of diabetes and/or hypertension. In the present population and independent of these clinical conditions, the increment of UAE was weakly associated to genotypes of SNPs located in the chromosomes 11, 12 and 16, replicating previous studies. These SNPs were located in genes such as G protein beta polypeptide 3, *ACEI* and *RPH3A*, associated previously to UAE [7] and to metabolic pathways not previously associated with UAE. However, the degree of association was not high enough to be considered as a positive association *per se*. Then we used the data from the metabolomic study to gain further insight into the potential relationship between genotypes and microalbuminuria.

A characteristic metabolomic profile associated to microalbuminuria was identified by using a multivariate model, which allows for discrimination between normoalbuminuric and microalbuminuric individuals. The good match between the results in training and cross-validation datasets provides further support to the model. Whereas previous studies reported correlations between metabolic profile and different CVD risk factors and disease states such as insulin resistance, diabetes, obesity [22], the present study represents the first description of metabolic profiles of microalbuminuria in a general population. The differential metabolic profiles show that branched amino acids (BCAA) are reduced in microalbuminuria. The statistical significance of different spectral regions containing resonances of BCAA and related metabolites, like 3-OH-isovalerate, supports the association. BCAA can act as signaling molecules in many processes. Although many studies report increased BCAA levels in diabetes and insulin resistance, the association with microalbuminuria has not been previously explored. Early studies showed that idiopathic portal hypertension correlates to decreased levels of leucine, isoleucine and valine [23]. Diet-induced insulin resistant obese mice also display a depletion of BCAA serum levels [24]. The interpretation of these findings is complex because fasting status, diet, exercise and basal metabolism affect BCAA levels in diverse ways [25]. The combined effect of lipids and BCAA seems pivotal in a complex network of interactions involving muscle, adipose, liver and brain metabolisms [26].

The microalbuminuric pattern, mainly in hypertension and/or diabetes, was also associated to alterations in glucose metabolism, lipid β-oxidation and the tricarboxilic acid (TCA) cycle [27], [28]. These are central metabolic cores for all eukaryotic cells. We report changes in lipids, glucose, pyruvate, lactate, alanine and glutamine which suggest important shifts in energy metabolism. However, the interpretation of these changes in relation to develop microalbuminuria is unclear. Different studies reported changes in different directions for these metabolites in obesity and related complications [29]. In the present study, glutamine, the most abundant amino acid in plasma, is also associated to microalbuminuria. Glutamine can be produced in the TCA cycle via 2-oxoglutarate and glutamate. It is also an important precursor of urea. As a consequence, glutamine plays a pivotal metabolic role, which can be affected by alterations in both TCA cycle and urea metabolism. Finally, choline, containing compound resonances associated to microalbuminuria, was also observed. Although choline is involved in multiple metabolic pathways, it has a predominant role in cell membrane integrity, methyl metabolism and lipid-cholesterol transport [30].

Although the correlation between rs10492025 and rs4359, and microalbuminuria is only suggestive in our study, the fact that patients with genotypes associated to an increase in UAE shared a similar metabolomic profile in the normoalbuminuric and in those with microalbuminuria, points to the presence of similar underlying mechanisms that predispose to develop an increase of UAE. The metabolomic profile characteristic of the subjects with microalbuminuria was observed also in normoalbuminuric subjects with the allele of risk or protection, in two of the polymorphism analyzed. These two polymorphisms are linked to the *ACE-I* and the *RPH3A* (rabphilin 3A homolog) genes. While the *ACE-I* gene has been very well characterized for many years and associated to UAE [31] and to progression of renal diseases [32], the role of the RPH3A has not been clarified until recently [33]. RPH3A is a RAB3A effector, small G protein that is thought to act at late stages of exocytosis. This small protein, present in neurons and in podocytes, confers specificity to vesicles. It is noteworthy that podocytes possess Rab3A and their specific effector rabphilin-3A, which is expressed only in cells capable of highly regulated exocytosis [34]. It is well known that podocytes are involved in many glomerular functions and apart from the maintenance of the filtration barrier they are responsible for the turnover of glomerular basement membrane components and for the ability to produce a variety of cytokines and growth factors [34]. Furthermore, in human proteinuric conditions, the expression of these molecules can increase, supporting the concept that the Rab3A-rabphilin-3A complex can play a role not only in normal but also in damaged podocytes, and consequently filtering albumin.

The study should be considered within its limitations since it is a cross-sectional study, in which associations can result from chance, and no cause-effect can be assured. The sample size is small for a genetic association study in complex trait genetics and some of the reported associations may be by chance. Concerning the correction for multiple testing, it reduces the significance of the association, but considering: a) each measurement has been independently inquired through both unbiased high-throughput genomics and metabolomics; thus, the integrative strategy of both techniques allows combination of the strength and compensates for the limitations on each of the methods; b) the SNP selection was from previous studies with a positive association with UAE, and therefore the present can be considered as a replication study; c) the two polymorphisms, in the genes ACE-I and the RPH3A, have a similar “metabolomic microalbuminuric profile” we consider that all of this reduce the possibility that the association was by chance.

## Perspectives

Subjects sharing genotype and metabolomic profile but discordant in UAE have different characteristics that are opposite in the two associated polymorphisms. While the TT genotype of the rs10492025 polymorphism of the *RPH3A* increases the risk, the CC genotype of the rs4359 polymorphism of the *ACE-I* gene reduces it. The microalbuminuria subjects were an average of 17 years younger in the former, and 7 years older in the latter genotypes. We hypothesize that these genetic backgrounds of *RPH3A* and *ACE-I* interact with the BP values and/or the presence of diabetes for the risk to develop microalbuminuria. The study is exploratory and hypothesis-generated. Then prospective studies need to confirm or refute this hypothesis that may have important clinical implications.

The presence of underlying mechanisms, expressed by the metabolomic profile that can be recognized before an increase in UAE, will have clinical relevance with double the implication. First, previously unrecognized pathways can be involved in the development of an increment in UAE, a marker of endothelial dysfunction and the starting point of many cardiovascular and renal diseases. The possibility to identify new mechanisms involved in the increment in UAE opens the possibility to develop targets to prevent or delay cardiovascular disease. Second, the metabolomic fingerprint may allow the identification of subjects at risk to develop microalbuminuria and consequently to have high CV and renal risk. Early identification of subjects at risk to develop an increase in UAE can be carried out even before the development of the classical cardiovascular risk factors, allowing for the reinforcement of preventive actions. The interaction of these mechanisms with the genetic background can allow for the identification of the precise risk and consequently to move towards a more personalized intervention in the high-risk subjects.

## Supporting Information

Figure S1 **PCA model scores plot for discrimination between patients without (open circles) and with microalbuminuria (close circles) based on the NMR spectra of blood serum of the entire cohort.**
(TIF)

Table S1 **General characteristics of subjects with normoalbuminuria and microalbuminuria for the two genotypes (rs4359_CC and rs10492025_TT).**
(DOCX)
