# Supplementary figures and images for: Genomic and Metabolomic Profile Associated to Microalbuminuria
Source: PLoS One. 2014 Jun 11;9(6):e98227. doi: 10.1371/journal.pone.0098227 (PMC4053470; doi:10.1371/journal.pone.0098227)

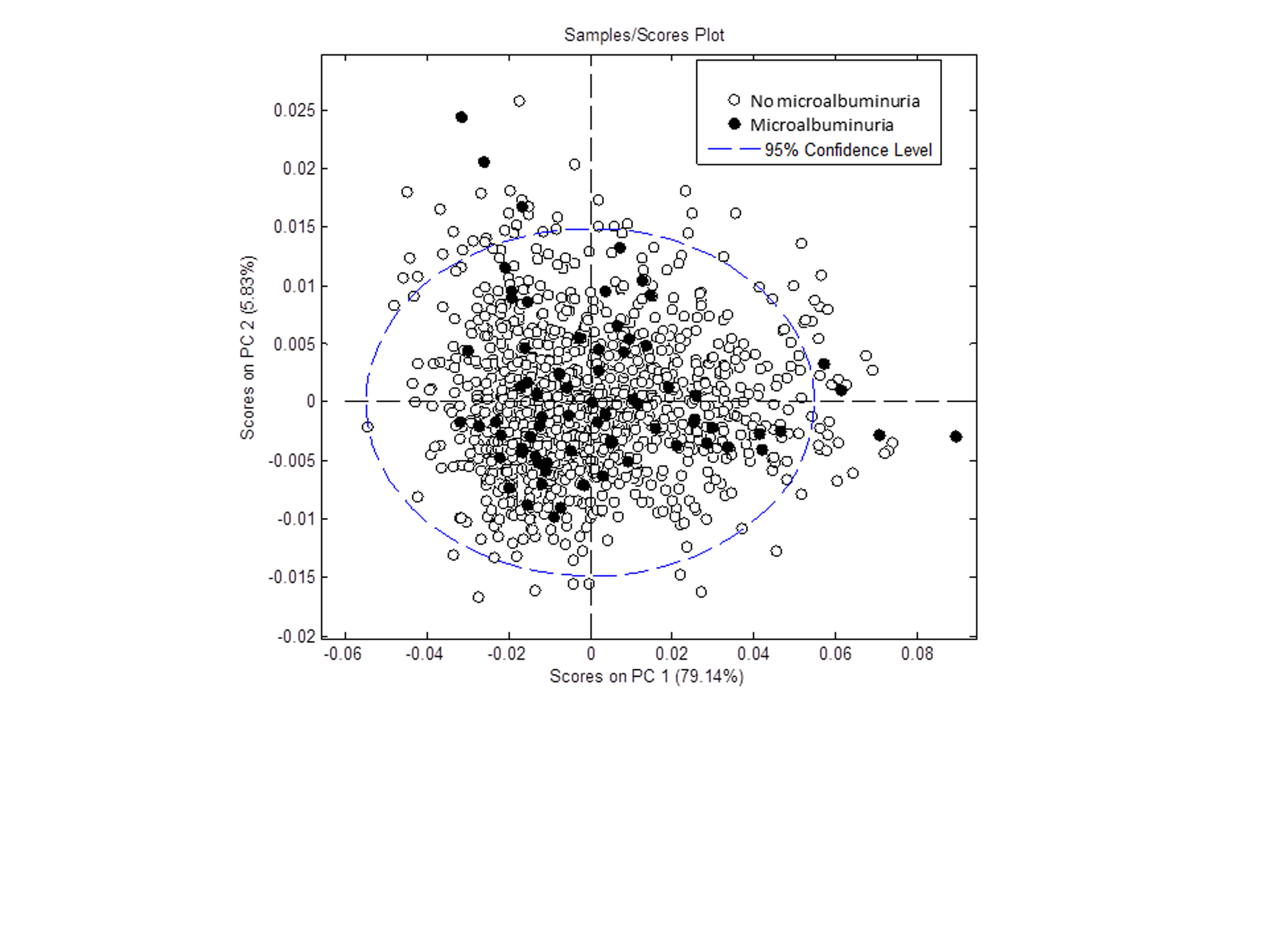

Supplement: Figure S1 — PCA model scores plot for discrimination between patients without (open circles) and with microalbuminuria (close circles) based on the NMR spectra of blood serum of the entire cohort. (TIF) [file pone.0098227.s001.tif]
