# Supplementary material for: Genomic and Metabolomic Profile Associated to Microalbuminuria
Source: PLoS One. 2014 Jun 11;9(6):e98227. doi: 10.1371/journal.pone.0098227 (PMC4053470; doi:10.1371/journal.pone.0098227)
Supplement: Table S1 — General characteristics of subjects with normoalbuminuria and microalbuminuria for the two genotypes (rs4359_CC and rs10492025_TT). (DOCX) [file pone.0098227.s002.docx]

**Table S1.**

**rs4359_CC rs10492025_TT**

|  | No Malb | Malb | p | No Malb | Malb | p |
| --- | --- | --- | --- | --- | --- | --- |
| Number of samples | 169 | 17 |  | 117 | 7 |  |
| Sex (M/F) | 86 / 83 | 6 / 11 | 0.309 | 57/ 60 | 4 /3 | 0.44 |
| Age (years) | 52 ± 19 | 74 ± 8 | <0.001 | 50 ±18 | 57 ±16 | 0.30 |
| BMI (kg/m^2^) | 26 ± 4 | 29 ± 6 | 0.020 | 26 ± 4 | 29 ± 5 | 0.122 |
| SBP (mmHg) | 129 ± 19 | 155 ± 22 | <0.001 | 127 ± 20 | 152 ± 13 | 0.004 |
| DBP (mmHg) | 78 ± 10 | 87 ± 14 | 0.002 | 78 ± 10 | 91 ± 9 | 0.003 |
| Glycemia (mg/dl) | 94 ± 22 | 105 ± 20 | 0.038 | 91 ± 17 | 95 ± 13 | 0.59 |
| Creatinine (g/24h) | 115 ± 59 | 125 ± 87 | 0.56 | 110 ± 60 | 121 ± 78 | 0.66 |
| Total Cholesterol (mg/dl) | 204 ± 33 | 221 ± 38 | 0.046 | 203 ± 35 | 202 ± 37 | 0.92 |
| LDL (mg/dl) | 116 ± 30 | 130 ± 39 | 0.093 | 113 ± 32 | 103 ± 27 | 0.45 |
| HDL (mg/dl) | 52 ± 13 | 49 ± 13 | 0.297 | 53 ± 14 | 54 ± 13 | 0.80 |
| LogTG (mg/dl) | 2,2 ± 0,2 | 2,3 ± 0,2 | 0,032 | 2.20±0.24 | 2.32± 0.20 | 0.25 |
| Diabetes Mellitus 2 | 12 (7.1%) | 5 (29.4%) | 0.011 | 8 (6.8%) | 2(33.3%) | 0.075 |
| Hypertension | 65 (38.5%) | 15 (88%) | <0.001 | 43(36.8%) | 5 (83.3%) | 0.033 |
| Metabolic Syndrome | 39 (23%) | 12 (71%) | <0.001 | 23 (19.7%) | 2(33.3%) | 0.60 |
| Obesity | 43 (26.1%) | 8 (47.1%) | 0.088 | 18 (15.8%) | 3(50%) | 0.066 |

*( ) percentage*
